# Clinical and serological evaluation of a novel CENP-A peptide based ELISA

**DOI:** 10.1186/ar3029

**Published:** 2010-05-20

**Authors:** Michael Mahler, Liesbeth Maes, Daniel Blockmans, Rene Westhovens, Xavier Bossuyt, Gabriela Riemekasten, Sandra Schneider, Falk Hiepe, Andreas Swart, Irmgard Gürtler, Karl Egerer, Margrit Fooke, Marvin J Fritzler

**Affiliations:** 1 Dr. Fooke Laboratorien, Mainstrasse 85, 41469 Neuss, Germany; 2 Current address: Department of Immunopathology, INOVA Diagnostics, San Diego, CA 92131-1638, USA; 3 Laboratory Medicine, General internal medicine, Rheumatology, University Hospitals Leuven, Binkomstraat 2 3210 Lubbeek, Belgium; 4 Charité University Hospital, German Rheumatism Research Centre, a Leibniz institute, Dept Rheumatology and Clinical Immunology Charitéplatz 1, 10117 Berlin, Germany; 5 Rheumatology Clinical Neuss, Neuss 41460, Germany; 6 Interdisziplinäres Autoimmun-Speziallabor, Charité University hospital, Campus Virchow-Klinikum, Augustenburger Platz 1, 13353 Berlin, Germany; 7 Faculty of Medicine, University of Calgary, 3330 Hospital Dr NW, Calgary, Alberta, T2N 4N1, Canada

## Abstract

**Introduction:**

Anti-centromere antibodies (ACA) are useful biomarkers in the diagnosis of systemic sclerosis (SSc). ACA are found in 20 to 40% of SSc patients and, albeit with lower prevalence, in patients with other systemic autoimmune rheumatic diseases. Historically, ACA were detected by indirect immunofluorescence (IIF) on HEp-2 cells and confirmed by immunoassays using recombinant CENP-B. The objective of this study was to evaluate a novel CENP-A peptide ELISA.

**Methods:**

Sera collected from SSc patients (n = 334) and various other diseases (n = 619) and from healthy controls (n = 175) were tested for anti-CENP-A antibodies by the novel CENP-A enzyme linked immunosorbent assay (ELISA). Furthermore, ACA were determined in the disease cohorts by IIF (ImmunoConcepts, Sacramento, CA, USA), CENP-B ELISA (Dr. Fooke), EliA^® ^CENP (Phadia, Freiburg, Germany) and line-immunoassay (LIA, Mikrogen, Neuried, Germany). Serological and clinical associations of anti-CENP-A with other autoantibodies were conducted in one participating centre. Inhibition experiments with either the CENP-A peptide or recombinant CENP-B were carried out to analyse the specificity of anti-CENP-A and -B antibodies.

**Results:**

The CENP-A ELISA results were in good agreement with other ACA detection methods. According to the kappa method, the qualitative agreements were: 0.73 (vs. IIF), 0.81 (vs. LIA), 0.86 (vs. CENP-B ELISA) and 0.97 (vs. EliA^® ^CENP). The quantitative comparison between CENP-A and CENP-B ELISA using 265 samples revealed a correlation value of rho = 0.5 (by Spearman equation). The receiver operating characteristic analysis indicated that the discrimination between SSc patients (n = 131) and various controls (n = 134) was significantly better using the CENP-A as compared to CENP-B ELISA (*P *< 0.0001). Modified Rodnan skin score was significantly lower in the CENP-A negative group compared to the positive patients (*P *= 0.013). Inhibition experiments revealed no significant cross reactivity of anti-CENP-A and anti-CENP-B antibodies. Statistically relevant differences for gender ratio (*P *= 0.0103), specific joint involvement (Jaccoud) (*P *= 0.0006) and anti-phospholipid syndrome (*P *= 0.0157) between ACA positive SLE patients and the entire SLE cohort were observed.

**Conclusions:**

Anti-CENP-A antibodies as determined by peptide ELISA represent a sensitive, specific and independent marker for the detection of ACA and are useful biomarkers for the diagnosis of SSc. Our data suggest that anti-CENP-A antibodies are a more specific biomarker for SSc than antibodies to CENP-B. Furthers studies are required to verify these findings.

## Introduction

Anti-centromere antibodies (ACA) have been repeatedly demonstrated to be useful biomarkers in the diagnosis of systemic sclerosis (SSc) in that they occur in 20 to 40% of these patients and are most commonly associated with the limited cutaneous subset (lSSc) of the disease, also known as the CREST (calcinosis, Raynaud's phenomenon, esophageal dysmotility, sclerodactyly, and telangiectasia) syndrome [1]. Although ACA are relatively specific for SSc, they have also been reported in systemic lupus erythematosus (SLE), primary biliary cirrhosis (PBC), rheumatoid arthritis (RA), Sjögren Syndrome (SjS), Raynaud's phenomenon and in subjects with no apparent connective tissue disease [2-11]. Although a number of CENP proteins, CENP-A, -B, -C, -D,-E, -F, -G, -H -O, have been described [1,12-14], CENP-A, -B and - C are thought to be the major targets of the anti-CENP immune response [1,2,15,16].

Historically, ACA were detected by indirect immunofluorescence (IIF) on HEp-2 cells and then confirmed by immunoassays that utilized recombinant CENP-B [17-19]. This protein, cloned in 1987 by Earnshaw et al., was eventually expressed as a eukaryotic recombinant protein and then adapted in an ELISA for autoantibody (aab) detection [18-22]. Similarly, the CENP-A protein was also cloned and a recombinant protein used for the detection of ACA by ELISA [23,24]. Despite these advances, only a few commercial diagnostic kits used the recombinant CENP-A protein because it has been assumed that CENP-B was the major autoantigen reactive with SSc sera [18]. Furthermore, while IIF is widely used as a screening test for ACA, it was reported that only sera with anti-CENP-B reactivity showed the typical CENP IIF staining pattern on HEp-2 cells [6,7]. This raised the question of the potential clinical value of alternate methods to screen for ACA in SSc and other conditions. In a recent study, it was found that the anti-CENP immune response differed between patients with SSc and SjS: 7/10 (70%) of SjS patients with CENP aabs recognized CENP-C alone compared to 1/18 (6%) SSc patients (*P *= 0.003). CENP-H antibodies have also been described as a biomarker for SjS as evidenced by their reactivity in 17/62 patients [14]. Consequently, these and related studies suggest that it may be clinically important to test for the individual CENP components as a diagnostic approach to differentiate between different diseases [6,7].

CENP-A is a 17 kDa protein that shares high sequence identity and homology with histone H3. In this context, it has been speculated that IgM aabs from a subset of patients with undifferentiated rheumatic disease syndromes staining mouse kidney nuclei with a distinctive variable large-speckled (VLS) IIF pattern recognized an epitope on histone H3 and a (H3-H4)2 tetramer, and since these sera also stained centromeric heterochromatin, it was suggested that they bound CENP-A [25]. Only a few groups have published studies on CENP epitopes [26-29]. In 2000, two groups independently analysed the epitope distribution on CENP-A which associated with active centromeres and has an important role in mediating the formation of specialized nucleosomes [27,30]. Using recombinant protein fragments as well as soluble and solid phase peptides, the anti-CENP-A immune response was shown to be directed against two domains in the N-terminus [26,27,30]. Within the two antigenic domains of CENP-A, a linear autoantigenic motif G/A-P-R/S-R-R that is repeated three times, was identified as the primary target of anti-CENP-A aabs. Of note, the epitope motif GPRRR is also present on CENP-B and CENP-C, which has been regarded as evidence for intra- and intermolecular epitope spreading that characterizes many other aab responses in systemic autoimmune rheumatic diseases [26-28]. Mimotopes of this motif were found in a number of other autoantigens and in the Epstein-Barr nuclear antigen 1 (EBNA-1) [28]. Using a similar approach, it was shown that some of the identified mimotopes cross-react with each other [28]. The observation that these mimotopes are cryptic epitopes may explain in part the obvious challenge of why CENP-A aabs retain high specificity. In a longitudinal study of a SSc patient, it was shown by ELISA that aab reactivity to CENP-A can be induced by intra- and inter-molecular epitope spreading from histone H3 and that antibodies to CENP-A peptides can temporally precede autoreactivity to recombinant CENP-B [31]. CENP-A derived peptides thus represent interesting tools for the diagnosis of early SSc. The potential of CENP-A peptides as diagnostic analytes was recently demonstrated in a study of ACA positive SSc patients [29]. The aim of the present study was the development and clinical characterization of a CENP-A peptide based ELISA in an international multi-centre study.

## Materials and methods

### Sera

Clinically defined sera were collected from SSc patients and various controls including SLE, RA, mixed connective tissue disease (MCTD), other disease conditions and healthy donors (HD) (for details see below). Patients with SSc were diagnosed according to criteria as described previously [32]. All other patients with autoimmune disorders were classified according to the published criteria for each disease as also applied in a recent investigation [33]. Patient data were anonymously used under consideration of the latest version of the Helsinki Declaration of human research ethics. Collection of patient samples was carried out according to local ethics committee regulations and where required written approval was obtained from the respective Institutional Review Board. The study was carried out according to the Directive 98/79/EC of the European Parliament and of the Council and the German law for medical devices (Medizinproduktegesetz). Due to the retrospective study design using stored samples only, patient consent was not required.

Samples derived from four different clinical centres including Charité University of Medicine, Department of Rheumatology and Clinical Immunology (Berlin, Germany), Rheumatology Clinic Dr. Gürtler (Neuss, Germany) and Laboratory Medicine, Immunology, University Hospitals Leuven (Leuven, Belgium) and the University of Calgary (Calgary, Canada) were tested for anti-CENP-A aabs by ELISA (Dr. Fooke Laboratorien). In addition, the Centers for Disease Control and Prevention (CDC) anti-nuclear antibody (ANA) reference sera [34] were analysed. Sera were stored at -20°C until use.

Patients at Charité University of Medicine, Department of Rheumatology and Clinical Immunology were diagnosed and characterized as recently described [35] and this cohort was used for the evaluation of serology-clinical associations. Autoantibodies to CENP-B, Scl-70 (topoisomerase I; topo-I), U1-RNP, SS-A/Ro, SS-B/La and Sm were determined using the EliA^® ^System on the UniCAP100^® ^(Phadia, Freiburg, Germany). Anti-Ro52 and anti-Ro60 antibodies were measured using the EUROLINE-Westernblot (ANA-Profil 3, Euroimmun AG, Lübeck) by automated incubation (EUROBlotMaster, Euroimmun AG) and computer based interpretation of results (EUROLineScan, Euroimmun AG). For the evaluation of fibrotic skin changes, the modified Rodnan Skin Score (mRSS) was used [36]. Anti-PM/Scl antibodies were detected by PM1-Alpha ELISA (Dr. Fooke, Laboratorien GmbH, Neuss, Germany).

### Peptides and protein antigens

The N-terminal sequence of human CENP-A was used as the template for the commercial synthesis of synthetic peptides (Peptides & Elephants, Berlin, Germany). The quality and purity of the peptide were assessed by mass spectrometry and analytical high-performance liquid chromatography. Recombinant CENP-A and CENP-B expressed in insect cells were obtained from a commercial supplier (Diarect AG, Freiburg, Germany).

### Autoantibody assays

IIF was performed on HEp-2 substrate kits (HEp-2000; ImmunoConcepts, Sacramento, CA, USA) that included fluorescein-conjugated goat antibodies to human IgG (H+L) in Calgary (Mitogen Advanced Diagnostics Laboratory, Calgary, AB, Canada). IIF patterns were read at serum dilutions of 1:160 and 1:640 on a Zeiss Axioskop 2 plus (Carl Zeiss, Jena, Germany) fitted with a 100-watt USHIO super-high-pressure mercury lamp (Ushio, Steinhöring, Germany) by two experienced technologists with more than five years of experience but who had no knowledge of the CENP-A ELISA results. CENP-B ELISA (REF: 25004, Dr. Fooke Laboratorien GmbH) with recombinant full-length CENP-B expressed in insect cells was used and performed according to manufacturer's instructions.

### ELISA with recombinant CENP-A and synthetic CENP-A peptide

Recombinant CENP-A antigen expressed in insect cells was purchased from a commercial supplier (Diarect AG) and coated at different concentrations ranging from 0.2 μg/mL and 0.4 μg/mL and in different buffers onto mircotiter plates (Maxisorb, Nunc, Denmark). The best discrimination between positive and negative controls was observed at a coating concentration of 0.2 μg/mL. This setup was used for further experiments. The CENP-A ELISA (Dr. Fooke Laboratorien GmbH), a CE-certified peptide based assay, was performed according to the manufacturer's AI-Line instructions for use. Briefly, the ELISA plates were prepared by determining the optimal CENP-A peptide concentration, followed by coating ELISA plates (Maxisorb; Nunc) overnight with 0.4 μg/well of the peptide in phosphate-buffered solution (pH 7.6). Non-specific binding sites were blocked by incubating in 0.5% bovine serum albumin in phosphate-buffered saline for 30 minutes. Calibration of the test was achieved using a positive control sample. The relative units were calculated by dividing the mean value of the optical densities (OD) of each patient by the mean OD value of the calibrator and multiplied by a conversion factor. All of the serum samples, the positive and negative controls, were tested in duplicate. In assay applications, sera were diluted 1:100 in Sample Buffer and then incubated for 30 minutes at room temperature. Following a washing step, anti-human IgG horseradish peroxidase was added and incubated for an additional 30 minutes. TMB Substrate was added after another washing step and the reaction was stopped.

Two different line immunoassays (LIAs) were used to test ANA reference sera (CDC) for their aab profiles. The first LIA (recomLine ENA/ANA IgG, Mikrogen GmbH, Neuried, Germany) contained the autoantigens: RNP68, RNP-A, RNP-C, SmB, SmD, SSA/Ro60, Ro52, La/SSB, Rib-P, PCNA, CENP-B, Scl-70, Jo-1, histone and dsDNA. The second LIA, the INNO-LIA™ ANA (Innogenetics, Gent, Belgium) contained the autoantigens: SmB, SmD, RNP-70k, RNP-A, RNP-C, Ro52, SS-A/Ro60, SS-B/La, CENP-B, topo-I/Scl-70, Jo-1, ribosomal P, histones.

### Inhibition assays

Serum samples were selected that showed comparable results in CENP-A and CENP-B ELISA and diluted in Dilution Buffer to obtain a reactivity of approximately 1.0 OD under standard assay conditions. Increasing concentrations of CENP-A derived peptide or recombinant CENP-B were added to the diluted sera and incubated for two hours at room temperature. Specimens were then assayed in CENP-A and CENP-B ELISA.

### Statistical evaluation

The data were statistically evaluated using the Analyse-it software (Version 2.03; Analyse-it Software, Ltd., Leeds, UK). A Wilcoxon-Mann-Whitney-U test was performed to analyze the differences between portions and Fisher exact test was used to analyze serology-clinical associations and associations of aabs. *P *values < 0.05 were considered as significant. *Kappa *(inter-rater agreement) and rank correlation (Spearmen correlation coefficient rho) were used to analyze putative agreement and correlation between groups. The strength of agreement with a kappa higher than 0.8 was considered as very good. Receiver-operating characteristic (ROC) analysis with area under the curve (AUC) evaluation was used to analyze the discrimination between different patient cohorts. Confidence interval (CI) was provided where appropriate.

## Results

### Anti-CENP-A antibodies measured by recombinant CENP-A and synthetic CENP-A peptides

Sera from patients with SSc and controls were tested for ACA using recombinant CENP-A, recombinant CENP-B or a synthetic CENP-A derived peptide. The ROC analysis indicated that the discrimination between SSc patients (n = 22) and various controls (n = 84) was significantly better using the CENP-A peptide ELISA than the CENP-B, or the recombinant CENP-A ELISA (data not shown). Although the differences between the AUC were statistically not significant (synthetic vs. recombinant *P *= 0.89), significant differences were observed in the sensitivity and specificity. At a cut-off value that corresponded to a specificity of 96.5%, 54.7% of the SSc patients were positive for anti-CENP-B and anti-CENP-A peptide antibodies, but only 22.7% for anti-CENP-A (recombinant protein). Based on this finding, the CENP-A peptide was used to detect CENP-A antibodies in further experiments.

### Qualitative comparison between CENP-A peptide ELISA and other methods for ACA detection

A total of 23/99 (23.2%) of the samples from SSc patients (Calgary cohort), tested for ANA by IIF, showed an ACA staining pattern on HEp-2 cells. The ROC analysis showed a good discrimination between IIF ACA positive and negative samples using the CENP-A ELISA (AUC = 0.86). The ELISA titre in the IIF positive group (mean = 7.9 relative units (RU)) was significantly higher than the IIF negative group (mean = 0.7 RU; *P *< 0.0001). The agreement according to the *kappa *calculation was 0.73 (*P *< 0.0001). One serum showed strong reactivity to CENP-A by ELISA and to CENP-B by LIA but did not have the classical ACA discrete speckled IIF staining pattern on interphase nuclei and metaphase chromatin. However, the serum did demonstrate speckled staining of interphase nuclei while staining of metaphase chromatin was absent, a pattern consistent with the previously described nuclear speckled pattern 1 (NSP-1) [17]. Another sample with weak reactivity for anti-CENP-A antibodies was negative by LIA (CENP-B) and also did not show the IIF ACA pattern. Very good agreement between the anti-CENP-A ELISA, CENP-B ELISA (*kappa *= 0.86) and LIA (*kappa *= 0.81) was found (Table 1).

**Table 1:** Qualitative agreement between CENP-A ELISA and other methods

n = 99^#^, kappa = 0.73	IIF		
CENP-A ELISA	pos (%)	neg (%)	Total
pos (%)	19 (19.2)	6 (6.1)	25 (25.3)
neg (%)	4 (4.0)	70 (70.7)	74 (74.8)
Total	23 (23.2)	76 (76.8)	99 (100.0)
			
n = 265*, kappa = 0.86^§^	**CENP-A ELISA**		
**CENP-B ELISA**	pos (%)	neg (%)	Total
neg (%)	4 (1.5)	205 (77.4)	209 (78.9)
Total	52 (19.6)	213 (80.4)	265 (100.0)
			
n = 100^#^, kappa = 0.81^§^	**LIA (CENP-B)**		
**CENP-A ELISA**	pos (%)	neg (%)	Total
pos (%)	20 (20.0)	5 (5.0)	25 (25.0)
neg (%)	2 (2.0)	73 (73.0)	75 (75.0)
Total	22 (22.0)	78 (78.0)	100 (100.0)
			
n = 100^#^, kappa = 0.88^§^	**IIF**		
**LIA CENP-B**	pos (%)	neg (%)	Total
pos (%)	19 (19.0)	3 (3.0)	22 (22.0)
neg (%)	1 (1.0)	77 (77.0)	78 (78.0)
Total	20 (20.0)	80.0	100 (100.0)
			
n = 82^#^, kappa = 0.97^§^	**EliA^® ^CENP-B**		
**CENP-A ELISA**	pos (%)	neg (%)	Total
pos (%)	32 (39.0)	1 (1.2)	33 (40.2)
neg (%)	0 (0.0)	49 (59.8)	78 (59.8)
Total	32 (39.0)	50 (61.0)	100 (100.0)

* SSc patients and controls; # SSc patients only; ^§^very good agreement

CENP: centromere protein; ELISA: enzyme linked immunoassay; IIF: indirect immunofluorescence; LIA: line-immunoassay

### Association between anti-CENP-A and anti-CENP-B reactivity by ELISA

The anti-CENP-A and anti-CENP-B titres of SSc samples (n = 131) and controls (n = 134) as measured by ELISA showed a clear correlation according to the Spearman analysis (rho = 0.5, CI 0.41 to 0.59, *P *< 0.0001). However, individual samples showed strong reactivity in only one assay. For example, one sample that was clearly positive by the CENP-A ELISA (7.0 RU) was negative by the CENP-B ELISA (0.5 RU). Vice versa, another serum clearly positive by the CENP-B ELISA (8.7 RU) was negative by the CENP-A ELISA (1.0 RU). The AUC calculated for the CENP-A ELISA in this cohort was significantly higher than for the CENP-B ELISA (0.81, CI 0.76 to 0.86 vs. 0.47, CI 0.39 to 0.55; difference 0.34, *P *< 0.0001). At a cut-off of 1.5 RU, the sensitivity and specificity was 36.6%/97.0% for CENP-A and 37.4%/94.8% for CENP-B (Table 2). The highest positive likelihood ratio was 49.1 at a cut-off of 1.75 RU for CENP-A and 44.0 at a cut-off of 3.5 RU for CENP-B (data not shown). When the two markers, namely anti-CENP-A and anti-CENP-B antibodies, were combined (CENP-A or CENP-B positive), the sensitivity increased to 38.2% at the expense of specificity which decreased to 93.1%. In contrast, when anti-CENP was defined as anti-CENP-A and anti-CENP-B positive, the sensitivity decreased to 35.9%, but the specificity increased to 99.3%.

**Table 2:** Prevalence of anti-CENP-A and anti-CENP-B autoantibodies by ELISA in different disease cohorts

	CENP-A				CENP-B				
	No. (%) >1.0 RU	No. (%) >1.5 RU	Mean/Median RU	Min/Max RU	No. (%) >1.0 RU	No. (%) >1.5 RU	Mean/Median RU	Min/Max RU	t-test CENP-B vs. A
SSc (n = 131)	64 (48.9)	48 (36.6)	2.37/0.98	0.1/7.9	53 (40.5)	49 (37.4)	2.77/0.47	0.2/9.6	*P *= 0.27
SLE (n = 109)	18 (16.5)	4 (3.7)	0.62/0.46	0.1/5.2	21 (19.3)	6 (5.5)	0.81/0.66	0.2/6.3	*P *= 0.027*
RA (n = 15)	0 (0.0)	0 (0.0)	0.29/0.26	0.1/0.5	0 (0.0)	0 (0.0)	0.51/0.49	0.3/0.9	*P *= 0.0002*
Other SARD (n = 10)	0 (0.0)	0 (0.0)	0.34/0.26	0.1/0.8	1 (10.0)	1 (10.0)	0.71/0.61	0.3/1.6	*P *= 0.024*
Controls all (n = 134)	18 (13.4)	4 (3.0)	0.56/0.43	0.1/5.2	22 (16.4)	7 (5.2)	0.76/0.61	0.2/6.3	*P *= 0.0047*

CENP: centromere protein; RA: rheumatoid arthritis; RU: relative units; SARD: systemic autoimmune rheumatic disease; SLE: systemic lupus erythematosus; SSc: systemic sclerosis

**Figure 1:**
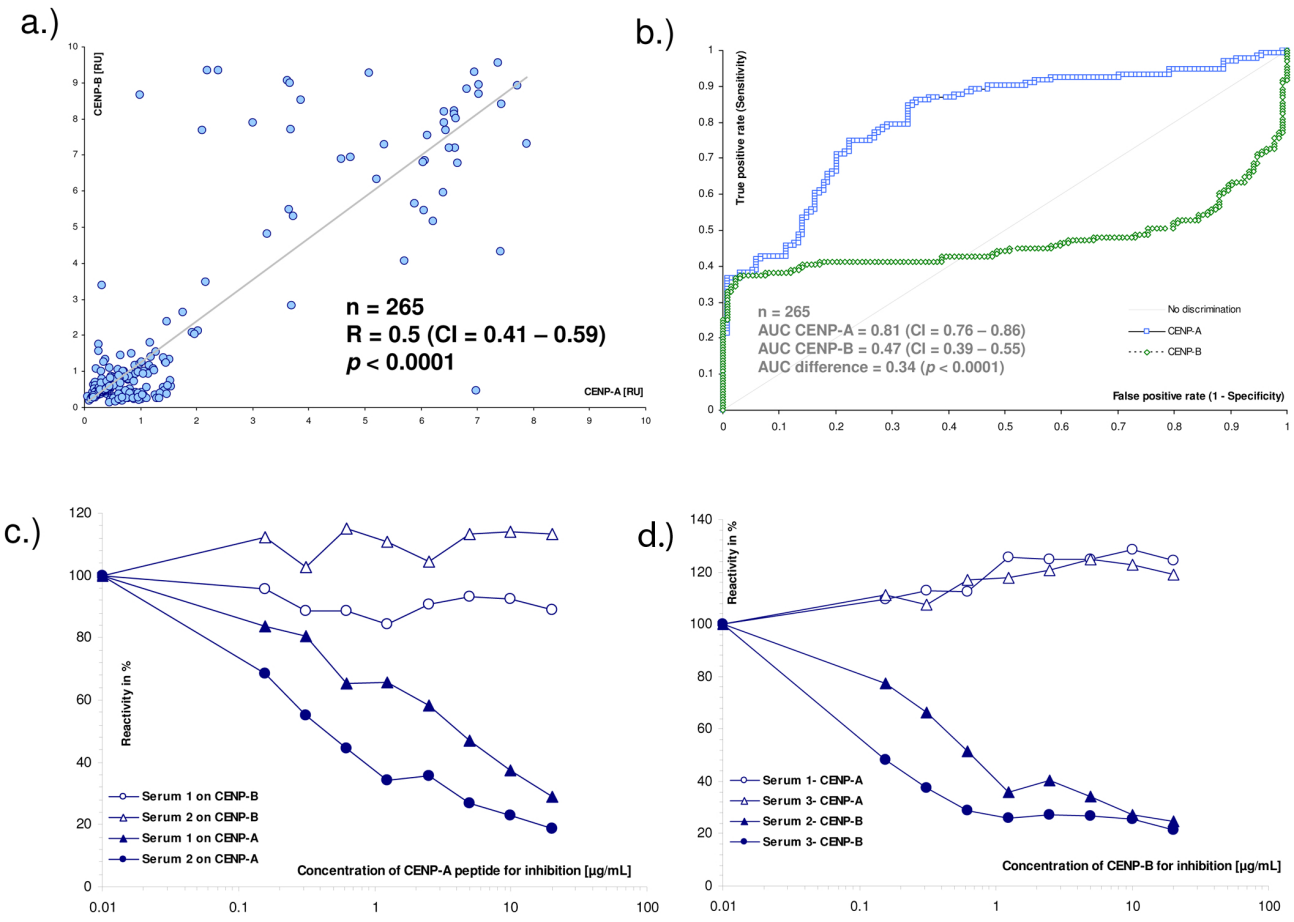
**Comparison between anti-CENP-A and anti-CENP-B reactivity**. Correlation diagram is shown in **a) **Comparative receiver operating characteristic analysis for the discrimination between SSc patients (n = 131) and controls (n = 134) is shown in **b)**. The area under the curve was significantly higher for CENP-A (0.81 vs. 0.47, *P *< 0.0001). Serum samples were selected that showed comparable results in CENP-A and CENP-B ELISA and diluted in Dilution Buffer to obtain a reactivity of approximately 1.0 OD. Increasing concentrations of CENP-A derived peptide and recombinant CENP-B were added to the diluted sera and incubated for two hours at room temperature. Specimens were then assayed in CENP-A and CENP-B ELISA. Inhibition was observed for anti-CENP-A reactivity with the CENP-A peptide **c) **and for anti-CENP-B reactivity with the recombinant CENP-B protein **d)**, but not with the respective other antigen.

Inhibition assays have shown that anti-CENP-A, but not anti-CENP-B reactivity can be blocked by pre-incubation of sera with soluble CENP-A derived peptide. As measured by ELISA, at a concentration of 20 μg/mL of the CENP-A peptide, the anti-CENP-A reactivity was reduced to <36% of the corresponding non-inhibited probe. Similar findings were observed vice versa, using CENP-B as inhibitor (see Figure 1c, d).

### Clinical sensitivity and specificity of the novel CENP-A peptide ELISA

**Figure 2:**
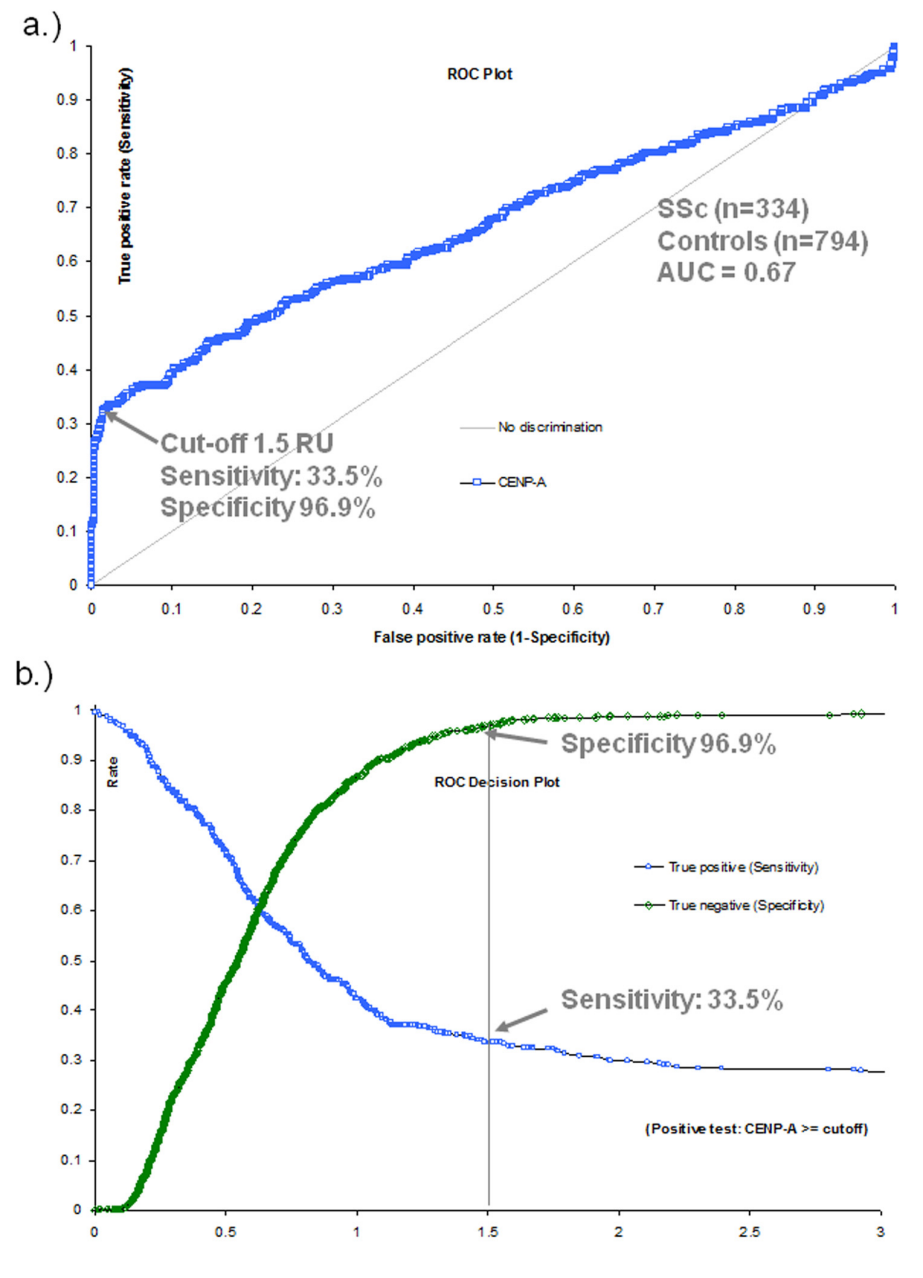
**Receiver operating characteristics analysis**. Receiver operating characteristics (ROC) analysis was performed using the data derived from all centres. Cut-off value of 1.5 RU is indicated by the arrows. ROC curve is shown in **a) **and ROC decision plot is shown in **b) **for the sensitivity and specificity.

**Figure 3:**
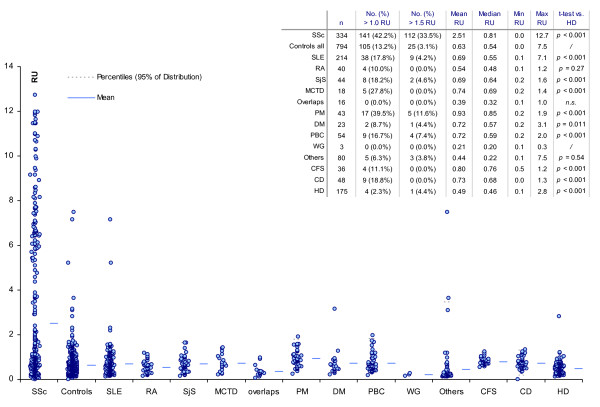
**Anti-CENP-A reactivity in different disease groups by comparative descriptive analysis**. The prevalence and titre of anti-CENP-A measured by CENP-A peptide ELISA was significantly higher in SSc patients compared to controls. Except for rheumatoid arthritis, the anti-CENP-A titres were significantly higher in all disease groups compared to the healthy donors.

Sera from 334 SSc patients and 794 controls from the participating centres were assayed for anti-CENP-A reactivity by ELISA (Table 3). By ROC analysis, a clear discrimination between SSc patients and controls was observed. The AUC value was found at 0.67 (CI 0.63 to 0.70, *P *< 0.0001) and at a cut-off of 1.5 RU the sensitivity was 33.5% and the specificity 96.9% (see Figure 2). The anti-CENP-A reactivity was significantly higher in SSc patients compared to the control group (*P *< 0.0001). When individual disease controls were compared to healthy donors, higher titres were found in most disease groups. No significant difference was observed between the individual disease control groups (see Figure 3).

**Table 3:** Multi-centre study of anti-CENP-A antibodies

	Berlin	Leuven	Calgary	Neuss	All
SSc	45/126 (35.7%)	30/84 (35.7%)	29/113 (25.7%)	8/11 (72.2%)	112/334 (33.5%)
lSSc	26/52 (50.0%)	26/29 (89.7%)	n.d.	n.d.	52/81 (64.2%)
dSSc	3/26 (11.5%)	3/29 (10.3%)	n.d.	n.d.	6/55 (10.9%)
Controls all	4/120 (3.3%)	9/226 (4.0%)	7/127 (5.5%)	5/321 (1.6%)	25/794 (3.1%)
SLE	4/109 (3.7%)	4/69 (5.8%)	n.d.	1/36 (2.8%)	9/214 (4.2%)
RA	n.d.	0/25 (0.0%)	n.d.	0/15 (0.0%)	0/40 (0.0%)
SjS	0/2 (0.0%)	2/35 (5.7%)	n.d.	0/7 (0.0%)	2/44 (4.6%)
MCTD	0/3 (0.0%)	0/12 (0.0%)	n.d.	0/3 (0.0%)	0/18 (0.0%)
Overlaps	0/4 (0.0%)	0/3 (0.0%)	n.d.	0/9 (0.0%)	0/16 (0%)
PM	n.d.	1/14 (7.1%)	4/28 (8.3%)	0/1 (0.0%)	5/43 (11.6%)
DM	n.d.	1/23 (4.4%)	n.d.	n.d.	1/23 (4.4%)
PBC	n.d.	n.d.	3/51 (5.9%)	0/3 (0.0%)	3/54 (5.6%)
WG	n.d.	n.d.	n.d.	0/3 (0.0%)	0/3 (0.0%)
Others	0/2 (0.0%)	n.d.	n.d.	3/78 (3.9%)	3/80 (4.6%)
CFS	n.d.	0/36 (0.0 %)	n.d.	n.d.	0/36 (0.0%)
CD	n.d.	n.d.	0/48 (0.0%)	n.d.	0/48 (0.0%)
HD	n.d.	1/9 (11.1%)	n.d.	0/166 (0.0%)	1/175 (0.6%)
					
Sensitivity	35.7%	35.7%	25.7%	n.d.	33.5%
Specificity	96.7%	96.0%	94.5%	98.1	96.9%.
AUC	0.80	0.68	0.42	n.d.	0.67

AUC: area under the curve; CD: Crohn's disease; CFS: chronic fatigue syndrome; DM: dermatomyositis; dSSc: diffuse cutaneous systemic sclerosis; HD: healthy donors, lSSc: limited cutaneous systemic sclerosis; MCTD: mixed connective tissue disease; n.d.: not determined; PBC: primary biliary cirrhosis; PM: polymyositis; RA: rheumatoid arthritis; SjS: Sjögren's syndrome; SLE: systemic lupus erythematosus; SSc: systemic sclerosis; WG: Wegener granulomatosis

In the entire SLE cohort, 9/214 (4.2%) tested positive for anti-CENP-A aabs. In a reduced SLE cohort tested for both anti-CENP-A and anti-CENP-B antibodies, 4/109 (3.7%) and 6/109 (5.5%) were positive, respectively. Although the reactivity to CENP-A and CENP-B was highly correlated (Spearman's rho = 0.84, *P *< 0.0001; kappa = 0.86), individual samples were either positive for anti-CENP-A or anti-CENP-B antibodies. Comparison of ACA (CENP-A or CENP-B) positive SLE patients with the entire SLE cohort revealed a statistically relevant increased prevalence of affected males (*P *= 0.0103), joint involvement (Jaccoud deformity) (*P *= 0.0006) and anti-phospholipid syndrome (*P *= 0.0157). When anti-CENP-B results were considered, a relevant difference was also found for liver involvement (*P *= 0.0313). The clinical features of ACA positive SLE patients are summarized in Table 4. No significant difference in the anti-CENP-A reactivity was observed in the SSc cohorts from the individual centres. The prevalence of anti-CENP-A antibodies in SSc was: 35.7% in Belgium, 35.7% in Germany (Berlin) and 25.7% in Canada and in good agreement with other methods for ACA detection used in the respective centres (Table 3). The clinical centre in Neuss, Germany was excluded from this analysis based on the limited number of SSc patients and the relatively high prevalence of patients with the limited skin form of SSc. Clinical data on skin involvement were available from 136 patients. In those, anti-CENP-A reactivity was significantly more prevalent in patients with limited cutaneous SSc compared to diffuse cutaneous SSc (*P *< 0.0001).

**Table 4:** Serology-clinical associations of anti-CENP antibodies in SLE

Patient ID	1	2	3	4	5	6	7	8	CENP pos CENP-A pos CENP-B pos	SLE cohort (n = 105)	
CENP-A/CENP-B [RU]	0.3/3.4	1.3/1.6	1.2/1.8	5.2/6.3	1.5/1.3	1.5/2.4	1.5/0.7	0.8/1.5	8/8 (100.0%)	8/105 (7.6%)	
CENP-A	neg	neg	neg	pos	pos	pos	pos	neg	4/8 (50.0%)	4/105 (3.8%)	
CENP-B	pos	pos	pos	pos	neg	pos	neg	pos	6/8 (75.0%)	6/105 (5.7%	
SLEDAI-2K	6	2	2	28	11	2	16	na	6	8	
Renal involvement	type V	no	yes*	type IV	type II	type V	no	type V	6/8 (75.0%) 3/4 (75.0%) 5/6 (83.3%)	70/105 (66.6%)	ns
Skin involvement	yes	no	no	no	no	no	yes	yes	3/8 (37.5%) 1/4 (25.0%) 2/6 (33.3%)	69/105 (65.7%)	ns
CNS involvement	no	no	no	no	no	no	no	yes	1/8 (12.5%) 0/4 (0.0%) 1/6 (16.7%)	13/105 (12.4%)	ns
Specific joint involvement (Jaccoud arthritis or deformity)	no	yes	yes	no	no	no	no	yes	3/8 (37.5%) 0/4 (0.0%) 3/6 (50.0%)	3/105 (2.9%)	*p *= 0.0006 ns *p *= 0.0002
Lung/heart involvement	no	no	yes	yes	no	no	yes	no	3/8 (37.5%) 2/4 (50.0%) 2/6 (33.3%)	37/105 (35.2%)	ns
Liver involvement	yes	no	no	no	no	no	no	yes	2/8 (25.0%) 0/4 (0.0%) 2/6 (33.3%)	4/105 (3.8%)	*p *= 0.0568 ns *p *= 0.0313
Anti-phospholipid syndrome	yes	yes	yes	yes	yes	yes	no	no	6/8 (75.0%) 3/4 (75.0%) 5/6 (83.3%)	31/105 (29.5%)	*p *= 0.0157 *p *= 0.1523 *p *= 0.0165
Raynaud's Syndrome	yes	no	no	no	no	yes	yes	yes	4/8 (50.0%) 2/4 (50.0%) 3/6 (50.0%)	54/105 (51.4%)	ns

* Specific disease type not known

CENP: centromere protein; CNS: central nervous system; na: not available; ns: not statistically significant; SLEDAI: Systemic Lupus Erythematosus Disease Activity Index

Note: Age and gender not included to protect anonymity of patients

Disease duration: mean, SLEDAI 2K: median

### Association of anti-CENP-A reactivity with other autoantibodies and clinical features

The mean age of anti-CENP-A positive group (n = 57) was 56.1 years (SD 12.5 years, maximum 82 years, minimum 27 years) and therefore not significantly different (*P *= 0.21) from the mean age of the anti-CENP-A negative group (n = 33) 52.7 years (SD 13.2 years, maximum 78 years, minimum 18 years). 35/38 (92.1%) of the anti-CENP-A negative group and 53/62 (85.5%) of anti-CENP-A positive group were female, with no significant difference between the groups. Rodnan skin score (RSS) was available for 90 SSc patients. The mean RSS was 5.5 (± 5.7; 95% CI 3.4 to 7.5) in the anti-CENP-A positive group (n = 33) and 8.9 (± 6.4; 95% CI 7.2 to 10.6) in the anti-CENP-A negative group (n = 57; *P *= 0.0090). Using the CENP-B ELISA, the mean RSS was 5.9 (± 5.6; 95% CI 3.9 to 8.0) in the anti-CENP-B positive group (n = 31) and 8.5 (± 6.6; 95% CI 6.8 to 10.2) in the anti-CENP-B negative group (n = 59; *P *= 0.0679).

Eighty-two sera from SSc patients were further analysed for co-occurrence of anti-CENP-A and other aabs (anti-Scl 70, -CENP, -U1-RNP, -SS-A/Ro60, -Ro52, -Ro60, -SS-B/La, -Sm). 33/82 (40.2%) samples were anti-CENP-A, 23/82 (39.0%) anti-Scl-70, 11/82 (13.4%) anti-U1-RNP and 9/82 (11.0%) anti-SS-A/Ro aab positive. None of the patients showed dual reactivity to CENP-A and Scl-70 or SS-A/Ro60. A significant difference between the CENP-A positive and negative group was found for anti-Scl-70 (*P *< 0.0001), anti-U1-RNP (*P *= 0.0429) and anti-SS-A/Ro (*P *= 0.0140), but not for anti-Ro52, anti-SS-A/Ro60, anti-SS-B/La, anti-Sm and anti-PM1-Alpha aabs (summarized in Table 5).

**Table 5:** Anti-CENP-A antibodies and other autoantibodies

Aab	CENP-A pos (n = 33)	CENP-A neg (n = 49)	All (n = 82)	Fisher's exact test *P*
Scl-70	0 (0.0%)	23 (46.9%)	23 (28.1%)	***P *< 0.0001**
U1-RNP	1 (3.0%)	10 (20.4%)	11 (13.4%)	***P *= 0.0429**
SS-A/Ro	0 (0.0%)	9 (18.4%)	9 (11.0%)	***P *= 0.0140**
Ro 52	8 (24.2%)	5 (10.2%)	13 (15.9%)	*P *= 0.1641
Ro 60	1 (3.0%)	0 (0.0%)	1 (1.2%)	*P *= 0.8049
SS-B/La	1 (3.0%)	4 (8.2%)	5 (6.1%)	*P *= 0.6523
Sm	0 (0.0%)	1 (2.0%)	1 (1.2%)	*P *= 1.0000
PM1-Alpha	1 (3.0%)	4 (8.2%)	5 (6.1%)	*P *= 0.6523

Aab: autoantibody; RNP: ribonucleoprotein

### Anti-CENP-A peptide reactivity in CDC ANA reference sera

As expected, only one of the 12 CDC reference sera (CDC 8, IS2134) was clearly positive for anti-CENP-A antibodies (2.7 RU). All other samples showed values < 0.4 RU. These findings are in good agreement with the results of the CDC reference laboratories [33] and with the LIA results (INNO-LIA™ ANA, recomLine ENA/ANA IgG: Mikrogen GmbH, Neuried, Germany).

## Discussion

ACAs are known to be reliable biomarkers and a clinically valuable adjunct in the prediction and diagnosis of SSc [37-39]. During the last two decades, recombinant CENP-B expressed in *E. coli *or insect cells had become the antigen of choice in immunoassays that were intended to confirm the presence of ACA reactivity initially identified by an IIF screening test on HEp-2 cells [1,17,22]. A controversy arose when individual studies reported ACA in diseases other than SSc such as SLE, SjS and PBC [2-4,6-10]. Although the prevalence of ACAs is 20 to 40% in SSc and only 3 to 5% in SLE, it has been suggested that numerically more patients with ACAs would actually suffer from SLE than from SSc because SLE is a much more prevalent condition than SSc. Further, it is well known that it is important to follow patients longitudinally for many years because one autoimmune condition can evolve to another [40] and several studies have reported overlap syndromes of SSc and other diseases such as SLE and RA [41]. Perhaps of more clinical importance, ACA have been reported to precede the diagnosis of SSc and have therefore been proposed as a biomarker to predict the onset of SSc [42,43]. Last but not least, the possibility of physician-based diagnostic error is also a potential complicating factor.

In our study, in some control groups the prevalence of anti-CENP-A aabs was lower than anti-CENP-B aabs, a finding in keeping with those of previous investigations [9]. Only 3/51 (5.9%) of PBC patients tested positive for anti-CENP-A, all of them exhibiting low antibody titres (<2 RU). Of note, previous studies have reported ACA reactivity in up to 60% of PBC patients by IIF (44%) and ELISA with recombinant CENP-B (60%) [2-4]. Remarkably, ACA were recently reported in 100% of SSc patients with co-morbid PBC [5]. Based on the data of the present study, one might speculate that ACA in diseases other than SSc are directed against CENP-B and perhaps other CENP antigens, rather than against CENP-A derived peptides. Further studies are needed to verify this observation.

It has been reported that antibodies affinity-purified from CENP-B are cross-reactive with CENP-A and vice versa [14]. In the cases where such cross-reactivity was not detected, N-terminally truncated CENP-B proteins were used, excluding the N-terminal GPKRR epitope [15]. In our study, antibody binding to CENP-B could not be blocked by absorption with the CENP-A derived peptide indicating that either the patients had anti-CENP-B antibodies that do not cross-react or that the cross-reactive aabs represents a minority of the polyclonal immune response to the CENP-B antigen. In addition, cross-reactivity cannot be excluded by the use of only one peptide for inhibition studies. However, CENP-A aabs seem to be independently expressed, but are closely related to the CENP-B aab system.

### Correlation with other methods

The results obtained with the novel CENP-A peptide ELISA exhibited good qualitative correlation with other methods for the detection of ACA, namely IIF on HEp-2 cells, LIA, ELISA and EliA™ CENP, the later three all using recombinant CENP-B. Further, the quantitative correlation between the ELISA results obtained with recombinant CENP-B and CENP-A peptide was moderate (rho = 0.5; *P *< 0.0001). These differences might be attributed to different epitopes recognized by anti-CENP-A and CENP-B antibodies. The observed agreements are in keeping with previous results. In a study by Sun et al., 95% of ACA positive samples (n = 38) reacted with recombinant CENP-A by ELISA and only 2/100 of ACA negative controls were anti-CENP-A positive [23].

In another study, the immune reactivity to CENP-A and CENP-B was analysed in detail [29]. ACA were identified by IIF on HEp-2 cells using a serum dilution of 1:360 as the cut-off. Sera showing an atypical centromere pattern, multiple IIF patterns, anti-dsDNA antibodies and/or detectable precipitating aabs to selected nuclear antigens (SS-A/Ro60, La, Sm, nRNP, Jo-1 and P) were excluded from the analysis. Of the 263 samples identified, 251/263 (95.4%) were positive on a commercial CENP-B ELISA (previously, Helix Diagnostics, Sacramento, CA, USA; now Bio-Rad, Hercules, CA, USA). A total of 246/263 (94%) of ACA positive samples targeted recombinant CENP-A in immunoblot and of these 7/263 (2.7%) reacted with CENP-A exclusively as detected by a CENP-A ELISA and immunoblot. Of those CENP-A positive samples, 197 (80%) reacted with multiple antigen peptide (MAP) peptide 2 (7 SRKPEAPRRRSPSP 20) and 219 (89%) with MAP peptide 3 (17 SPSPTPTPGPSRRG 30). Thus, 219/263 (83.3%) ACA positive samples reacted with MAP peptide 3. Moreover, the sensitivity of the MAP peptide ELISA described by Akbarali et al. was significantly lower than for the CENP-B ELISA (83.3% vs. 95.4%) [29]. The study by Akbarali et al. did not include an unselected cohort of SSc patients. Therefore, no information was obtained for the clinical sensitivity of the peptide based assays. Another limitation of the study was the lack of disease controls to prove the specificity of the peptide based ELISA assays [29]. Although we found no big difference between anti-CENP-A and anti-CENP-B aabs, the exclusive use of anti-CENP-B aab determination should not be the guideline for this autoantibody specificity. When anti-CENP-A and anti-CENP-B was combined the specificity significantly increased and the sensitivity remained almost unchanged. Therefore, combined or multiplexed testing for anti-CENP-A and anti-CENP-B or even for other ACA might further increase the diagnostic efficiency of autoantibody assays for ACA in the future.

Once a synthetic peptide has been identified as the target of aabs contained in sera of a defined cohort of patients suffering from a certain autoimmune disease, it represents an ideal antigen because it can easily be produced in high quality and quantity with minimal lot to lot quality variation (reviewed in [44]). Several studies have reported a higher sensitivity and/or specificity of peptide based immunoassays compared to assay systems with the respective native or recombinant antigen [44]. Examples of such peptides antigens are cyclic citrullinated peptides (CCP), a ribosomal P peptide called C22, the PM/Scl major epitope peptide PM1-Alpha and SmD derived peptides [44,45]. Whether CENP-A peptides will become widely used antigens for the detection of ACA need further investigation.

### Association of anti-CENP-A peptide reactivity with other autoantibodies and clinical features

Using newer diagnostic platforms, namely multiplex assay systems that provide a more detailed representation of the B cell response in an individual patient, it is very important to know which aabs coexist with ACA. This will hopefully lead to a clearer and more meaningful clinical understanding of concurrent or future comorbid autoimmune conditions in individual patients. As an example, it is well known that a proportion of patients with lcSSc may have or will eventually develop the autoimmune liver disease, primary biliary cirrhosis [46]. In that context, the presence of anti-CENP aabs coexisting with anti-mitochondrial aabs can help raise the awareness of the attending physician. Therefore, anti-CENP-A reactivity was considered in the context of other aabs. Antibodies targeting topoisomerase I (ATA or Scl-70) and ACA have historically been considered to be mutually exclusive. However, in a metanalysis of published studies, 28 cases of the coexistence of ACA and ATA were identified in 5,423 patients (0.52%) with SSc or SSc associated symptoms [46]. Therefore, the expression of ATA and ACA does not appear to be entirely mutually exclusive, although coincidence is rare (<1% of patients with SSc). Patients with both aabs often have diffuse SSc and show immunogenetic features of both aab defined subsets of SSc. In our smaller cohort, 0/23 ATA positive patients had ACA and did not react with CENP-B nor with CENP-A (*P *< 0.0001). It is important to revaluate such coincidences by using more modern and conventional technologies that are widely used in diagnostic laboratories today.

Of high interest, there was a significant association between ACA and the combined anti-SS-A analytes (Ro52 + SS-A/Ro60) but not when anti-Ro52 or anti-SS-A/Ro60 was tested as separate analytes. This finding, together with the observation that anti-Ro52 was more frequently detected as anti-SS-A (Ro52 + Ro60) and anti-Ro60, is consistent with the data published recently by Schulte-Pelkum et al. [47] where it was reported that anti-SS-A reactivity can be missed when Ro52 and SS-A/Ro60 antigens are combined in a single blended immunoassay.

### Clinical sensitivity and specificity of the novel CENP-A peptide ELISA

The sensitivity of 33.5% and specificity of 96.9% of the novel CENP-A peptide based ELISA is comparable to the characteristics published for ACA assays [1,2,5]. When CENP-A ELISA was directly compared to CENP-B ELISA, the former exhibited a better discrimination between SSc patients and controls as shown by ROC analysis (AUC = 0.81 vs 0.47; difference 0.34, *P *< 0.0001). Of note, a significant portion of the irrelevant AUC, at cut-off values below 1 RU, contributed to the difference in the AUC. However, at a cut-off of 1.5 RU, the sensitivity was almost equal (36.6% and 37.4%), but the specificity was higher for the ELISA CENP-A (97.0% vs. 94.8%).

Of interest, we found statistically relevant differences between ACA positive SLE patients and the entire SLE cohort. These included differences in the gender (for CENP-A or -B and for CENP-B), specific joint involvement (Jaccoud, for CENP-A or -B and for CENP-B), liver involvement (CENP-B) and anti-phospholipid syndrome (for CENP-A or -B and for CENP-B). In a previous study, cutaneous vasculitis, nodular vasculitis and Raynaud's phenomenon were associated with differences between ACA positive SLE patients [9], findings that were not found in our present study. The authors concluded that ACA can be detected in patients with genuine SLE without concurrent SSc, but that these patients do not represent a different clinical subgroup. However, a systematic approach in a large cohort of SLE patients is still required to analyse the clinical associations of ACA in SLE.

Although previous studies have demonstrated the applicability of recombinant CENP-A protein or CENP-A derived peptides for the detection of ACA [23,29], the present study is the first to describe putative advantages of an anti-CENP-A immunoassay, namely improved discrimination between SSc patients and controls. Since this is the first study to identify these differences, independent confirmation is important to clearly define the clinical usefulness of CENP-A based immunoassays.

## Conclusions

Anti-CENP-A antibodies measured by peptide ELISA are highly specific for SSc. Furthermore, the discrimination between SSc and patients and controls might be improved by the CENP-A ELISA compared to conventional methods for the detection of anti-CENP antibodies. In the serial steps of evaluation, different groups of SSc patients and control subjects were used, therefore a selection bias in our study cannot be completely excluded. Therefore, further studies are needed to verify these preliminary results.

## Abbreviations

Aab: autoantibody; ACA: anti-centromere antibodies; AUC: area under the curve; CCP: cyclic citrullinated peptides; CD: Chron's disease; CDC: center of disease control and prevention; CFS: chronic fatigue syndrome; CI: confidence interval; CREST: syndrome, calcinosis, Raynaud's phenomenon, esophageal dysmotility, sclerodactyly, and telangiectasia syndrome; DM: dermatomyositis; EBNA-1: Epstein-Barr nuclear antigen 1; HD: healthy donors; IIF: indirect immunofluorescence; LIA: line immunoassay; MAP: multiple antigen peptide; MCTD: mixed connective tissue disease; mRSS: modified Rodnan Skin Score; OD: optical densities; PM: polymyositits; PBC: primary billary cirrhosis; RA: rheumatoid arthritis; ROC: receiver-operating characteristic; RU: relative units; SARD: systemic autoimmune rheumatic disease; SSc: systemic sclerosis; SjS: Sjögren's syndrome; SLE: systemic lupus erythematosus; VLS: variable large-speckled; WG, Wegener granulomatosis.

## Competing interests

MM was employed at Dr. Fooke Laboratorien GmbH and is now employed at INOVA Diagnostics, Inc. MF is owner of Dr. Fooke Laboratorien GmbH selling the CENP-A ELISA. A patent application was submitted on the novel CENP-A ELISA. MJF is the Director of Mitogen Advanced Diagnostics Laboratory, Calgary, Alberta, Canada which provides diagnostic testing services and is paid an honorarium for consulting services to ImmunoConcepts Inc., Sacramento, CA, USA and INOVA. All other authors declare that they have no competing interests.

## Authors' contributions

MM and MJF take responsibility for the study design, analysis and interpretation of data, and manuscript preparation. MM and MJF had full access to all of the data in the study and take responsibility for the integrity of the data and the accuracy of the data analysis. Data acquisition was courtesy of MM, LM, RW, DB, XB, GR, KE, SS, FH, AS, IG and MJF. Statistical analysis was performed by MM.
